# Immunoregulatory Effect of Preventive Supplementation of Omega-3 Fatty Acid in a Complex Regional Pain Syndrome Type I Model in Mice

**DOI:** 10.3389/fnint.2022.818692

**Published:** 2022-03-22

**Authors:** Paula Franson Fernandes, Taynah de Oliveira Galassi, Verônica Vargas Horewicz, Afonso Shiguemi Inoue Salgado, Josiel Mileno Mack, Heloiza dos Santos Baldança, Ana Paula Ferreira da Silva, Stephen Bruehl, Edsel B. Bittencourt, Lynsey A. Seim, Daniel Fernandes Martins, Franciane Bobinski

**Affiliations:** ^1^Experimental Neuroscience Laboratory (LaNEx), Graduate Program in Health Sciences, University of Southern Santa Catarina, Palhoça, Brazil; ^2^Natural Quanta Wellness Center, Windermere, FL, United States; ^3^Graduate Program in Medical Sciences, Department of Medical Clinic, Federal University of Santa Catarina, Florianópolis, Brazil; ^4^Faculty of Medicine, University of Southern Santa Catarina, Palhoça, Brazil; ^5^Faculty of Physical Therapy, University of Southern Santa Catarina, Palhoça, Brazil; ^6^Department of Anesthesiology, Vanderbilt University Medical Center, Nashville, TN, United States; ^7^Coastal Health Institute, Jacksonville, FL, United States; ^8^Department of Hospital Internal Medicine, Mayo Clinic, Jacksonville, FL, United States

**Keywords:** omega-3, macrophages, complex regional pain syndrome, edema, cytokines

## Abstract

**Objective:**

Complex regional pain syndrome (CRPS) is usually triggered by trauma or a surgical procedure, and it typically becomes an established one after an intense inflammatory process with chronic pain and edema as the main symptoms. Available treatments for CRPS have low efficacy. This study aimed to evaluate the clinical and immunoregulatory effects of omega-3 polyunsaturated fatty acid (PUFA) supplementation on paw edema and anti- and pro-inflammatory cytokines and macrophage phenotypes in the chronic post-ischemia pain (CPIP) preclinical model of CRPS-Type I.

**Methods:**

Female Swiss mice were supplemented with omega-3, corn oil, or saline and then submitted to the CPIP model of ischemia/reperfusion (I/R) injury. Supplementation was carried out for 30 days prior to and up to 2 or 15 days after the induction of CPIP, according to experimental protocols. The supplementation protocol included 1,500 mg/kg of omega-3 or corn oil through an intragastric route (gavage). Paw edema, interleukin- (IL-) 4, IL-10, transforming growth factor-β1 (TGF-β1), monocyte chemotactic protein-1 (MCP-1), and tumor necrosis factor (TNF) were then measured in the paw skin and muscle by enzyme-linked immunosorbent assay (ELISA), and macrophage phenotypes (M1 and M2) assessed in the paw muscle by Western blotting.

**Results:**

The CPIP model induced an increase in paw thickness up to 72 h post-I/R. Mice supplemented with omega-3 compared to the saline group displayed reduced edema but neither altered skin IL-4 or skin and muscle TGF-β1, TNF, and MCP-1 concentrations, nor did they exhibit significantly altered muscle macrophage phenotype on the 2nd-day post-CPIP. However, omega-3 supplementation reversed the I/R-related reduction in IL-4 in the paw muscle compared to groups supplemented with saline and corn oil. Furthermore, omega-3 promoted the reduction of IL-10 levels in the paw skin, compared to animals with lesions supplemented with saline, until the 2nd-day post-CPIP. On the 15th day post-CPIP, IL-10 was significantly increased in the muscle of animals supplemented with omega-3 compared to the saline group.

**Conclusion:**

The results suggest that omega-3 PUFA supplementation has anti-inflammatory effects in the CPIP model of CRPS-Type I, significantly reducing paw edema and regulating concentrations of anti-inflammatory cytokines, including IL-4 and IL-10.

## Introduction

Complex regional pain syndrome (CRPS) occurs following trauma or surgical procedures and typically becomes an established one with an inflammatory process triggered by an injury. Affected individuals may present with autonomic, sensory, motor, and trophic clinical manifestations. Although regional pain is the main symptom, there are also significant vasomotor and sudomotor changes. In addition to allodynia and hyperalgesia, patients typically experience warm or cold limbs, altered sweating, edema, and an alteration in the appearance of skin and nails (Gaspar and Antunes, 2011; Beerthuizen et al., 2012; Field, 2013; Hauser et al., 2013; Birklein and Dimova, 2017; Russo et al., 2018).

The incidence of CRPS is higher in women aged 40–60 years old, and it predominantly affects the upper limbs (Sandroni et al., 2003; Birklein and Dimova, 2017), with the leading cause fracturing in the distal limbs (Sandroni et al., 2003; de Mos et al., 2007). Annual incidence is from 5.5 to 26.2 per 100,000 people at risk (Sandroni et al., 2003; de Mos et al., 2007). CRPS reduces the quality of life of affected individuals and is a burden on the healthcare systems. The chronic disabling pain of CRPS often causes withdrawal from work, social life, and impairments in basic daily activities (Birklein and Dimova, 2017).

Post-traumatic inflammation, vasomotor dysfunction, and neuronal plasticity characterize the pathophysiology of CRPS (Marinus et al., 2011). In patients with CRPS-Type I (CRPS occurring in the absence of major nerve injury), circulating concentrations of pro-inflammatory cytokines are increased, whereas anti-inflammatory cytokines are decreased (Schinkel et al., 2006; Uçeyler et al., 2007). Coderre et al. (2004) proposed that CRPS-Type I is a consequence of tissue damage due to ischemia and reperfusion, leading to the development of the chronic post-ischemia pain model (CPIP) in rats, which was subsequently adapted for mice by Millecamps et al. (2010). The CPIP model mimics some aspects of the presumed pathophysiology of CRPS, including increased microvascular vasoconstriction, tissue hypoxia, and metabolic acidosis. The CPIP model also produces several clinical features of CRPS, such as edema, increased paw temperature, allodynia, and mechanical and thermal hyperalgesia which can persist for up to 4 weeks (Bruehl, 2015). There is also evidence of increased neutrophil infiltration in the tissue 48 h post-injury. Consequently, from 2 to 48 h post-CPIP induction in rats, an increase is observed in inflammatory cytokines *via* the nuclear factor-κB (NF-κB) transcription factor pathways, such as tumor necrosis factor (TNF), interleukin- (IL-) 1β, and IL-6. Also, oxidative stress products present in the skin and muscle of the injured paw have been shown to contribute to perpetuating nociceptive sensitization and inflammation in the CPIP model (Laferrière et al., 2008; Xanthos and Coderre, 2008; Klafke et al., 2016).

After ischemia and reperfusion injury, the resident cells of the injured tissue—mainly macrophages—are activated and quickly release substances such as bradykinin, prostaglandins (PG), and prostacyclins, modulating the inflammatory process through vasodilation, increased vascular permeability, and plasma leakage (Huygen et al., 2001). Macrophages alter their phenotype and can exacerbate or repair the injury, depending upon the injured environment (Mantovani et al., 2004). The initial pro-inflammatory environment promotes the differentiation of M1 macrophages, characterized by increased expression of nitric oxide synthase 2 (NOS-2, also known as iNOS), which leads to a higher nitric oxide (NO) production (MacMicking et al., 1997; Arnold et al., 2014). M1 macrophages are considered pro-inflammatory and microbicidal, which are essential for host defense, although they can cause collateral damage to the inflamed tissue (Serhan et al., 2007).

In contrast, M2 macrophages are activated in the presence of anti-inflammatory cytokines such as IL-4 and IL-13 (Mantovani et al., 2004; Rőszer, 2015). The M2 macrophage phenotype expresses arginase-1, which inhibits NO production (Duluc et al., 2007; Rőszer, 2015) and promotes angiogenesis and remodeling of an extracellular matrix, thus, suppressing the inflammatory response (Komori et al., 2011). During the resolution phase, after the apoptosis of neutrophils, M2 macrophages interrupt the production of pro-inflammatory mediators such as TNF, IL-1β, and IL-6, and begin to release anti-inflammatory substances such as IL-10, transforming growth factor-β1 (TGF-β), and IL-1 receptor antagonist (IL-1ra) (Serhan et al., 2007).

Standard treatments used for managing CRPS currently include anti-inflammatory agents in the acute phase (e.g., corticosteroids), sympathetic nerve blocks, antidepressants, neuroleptics, and opioids, in addition to physical therapy, occupational therapy, and psychotherapy (Bruehl, 2015). Despite a wide variety of interventions employed and numerous experimental and clinical studies carried out on the topic, to date, there is no well-established and fully effective standard treatment for CRPS, and it remains a challenge for the medical and scientific community (Beerthuizen et al., 2012; Smart et al., 2016; Birklein and Dimova, 2017).

Omega-3 polyunsaturated fatty acids (PUFA, present in fish oil), particularly eicosapentaenoic acid (EPA) and docosahexaenoic acid (DHA), are the substrates for a family of lipid pro-resolving mediators called resolvins, protectins, and maresins, and are known for their anti-inflammatory (Serhan et al., 2008; Calder, 2015) and pro-resolving properties (Bannenberg and Serhan, 2010). Clinical studies have shown that EPA and DHA reduce the synthesis of pro-inflammatory cytokines such as IL-6, IL-1β, and TNF in inflammatory diseases. Also, omega-3 has been shown to mitigate the effects of inflammation, including leukocyte chemotaxis, the expression of adhesion molecules on the endothelium, and the production of eicosanoids, such as PG, thromboxanes, and leukotrienes, derived from arachidonic acid (Calder, 2008, 2015; Tan et al., 2018).

Considering the potential effects of omega-3, we hypothesized that preventive supplementation of fish oil rich in omega-3 may exert an immunoregulatory effect on the CRPS-related inflammatory process elicited by the CPIP model in female Swiss mice. The focus on females was due to the known higher incidence of CRPS in females (3:1 ratio relative to males) (Sandroni et al., 2003; de Mos et al., 2007; Birklein and Dimova, 2017) and sex differences in the development of the immune and behavioral pain responses in mice with CRPS (Guo et al., 2019). Specifically, we evaluated the anti-edematogenic effects of omega-3 supplementation and its potential for regulating pivotal anti-inflammatory cytokines, including IL-4, IL-10, and TGF-β1, as well as the pro-inflammatory cytokines TNF and monocyte chemotactic protein-1 (MCP-1). Also, we evaluated whether omega-3 supplementation would alter the macrophage phenotype present in mouse paw muscle in the CPIP model.

## Materials and Methods

### Animals and Ethical Aspects

Experiments were carried out using a total of 88 female Swiss mice (40–50 g, approximately 2 months old), obtained from the animal facility of the Federal University of Santa Catarina (Florianopolis, Brazil). The animals were housed in the Experimental Neuroscience Laboratory of the University of Southern Santa Catarina, in a room maintained at 22°C ± 2°C, on a 12-h light/dark cycle (lights on at 06:00), with access to food and water *ad libitum*. The animals were randomly distributed among the groups (described in section “Protocols, Experimental Groups, and Sample Calculation”) and allowed to adapt to the laboratory conditions for at least 1 h prior to the behavioral tests. All experimental protocols were approved by the appropriate Institutional Ethics Committee (CEUA, UNISUL, protocol no. 18.050.4.01.IV). The experiments were carried out under the Guide for Laboratory Animal Facilities and Care, Ethical Guidelines for Investigations of Experimental Pain in Conscious Animals (Zimmermann, 1983), ethical principles established by the National Council for the Control of Animal Experimentation (CONCEA), and with the legislation for the protection of animals used for scientific purposes (Directive 2010/63/EU revising Directive 86/609/EEC) and United States National Institutes of Health Guidelines.

### Induction of the Chronic Post-ischemia Pain Model

To induce the CPIP model, mice were anesthetized with an intraperitoneal (i.p.) injection of thiopental (80 mg/kg). After checking the state of unconsciousness, a 1.2-mm-diameter elastic ring was positioned proximal to the ankle joint of the animal’s right hind paw to make a tourniquet. The animal was kept anesthetized with the tourniquet for 3 h. Then, the elastic ring was cut, allowing the reperfusion of the paw. Anesthetic reinforcements were administered in those animals that returned from anesthesia before the stipulated period (thiopental, 20% of the initial dose) (Millecamps et al., 2010).

### Preventive Supplementation With Omega-3, Corn Oil, or Saline

For supplementation with omega-3 fish oil (Silva et al., 2017) or corn oil, a dose of 1,500 mg/kg [intragastric route (i.g.)] was used. For the group supplemented with saline (NaCl 0.9%) a volume of 50 μl (*via* i.g.) was used. The omega-3 supplement (in 1,000 mg capsules, containing 400 mg of EPA and 300 mg of DHA; Natural Quanta, Orlando, FL, United States) was removed from the original capsule using a syringe and immediately administered to animals *via* an i.g., Corn oil (Liza, Mairinque, SP, Brazil) was used as a supplementation control as it contains 50% omega-6 without known anti-inflammatory effects (and low levels of omega-3) and it is acceptable for daily use (Pérez et al., 2005; Freitas et al., 2016).

### Protocols, Experimental Groups, and Sample Calculation

**FIGURE 1:**
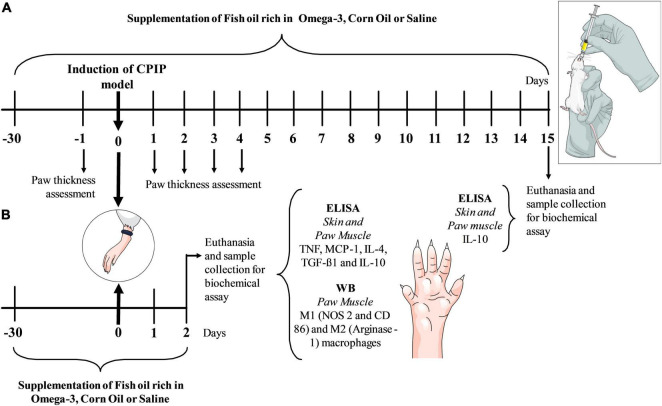
Experimental design with chronic omega-3 supplementation for 30 days and then baseline assessment of paw thickness with a micrometer (μm) before the ischemia and reperfusion procedure. Subsequently, the CPIP animal model was induced. The omega-3 supplements continued daily up to the 4th day after the induction of the model when daily assessments of paw thickness were performed. Following 15 days, structures were removed for an ELISA biochemical analysis **(A)**. In the second experiment, animals received the same supplementation protocol before the induction of the CPIP model and up to 2 days (48 h) after the induction of the model, where structures were removed for the biochemical ELISA and WB assays **(B)**. CPIP, Chronic Post-Ischemia Pain; WB, Western Blotting; ELISA, Enzyme Immunosorbent Assay.

Supplementation with omega-3, corn oil, or saline was carried out for 30 days prior to and up to 2 (protocol 2) or 15 days (protocol 1) after the induction of CPIP, according to experimental protocols (Figure 1). The measurement of hind paw edema thickness was performed 1 day prior to (baseline) and for 4 days after the induction of CPIP. In the first experimental protocol (Figure 1A), animals were euthanized (5% isoflurane) on the 15th day after the induction of CPIP and IL-10 concentrations in the paw skin and muscle were determined by enzyme-linked immunosorbent assay (ELISA). The second experimental set (Figure 1B) went through the same supplementation protocol with omega-3, corn oil, or saline but the animals were euthanized 48 h after the induction of CPIP. Mice were euthanized (5% isoflurane), and the paw skin and muscle were removed for the determination of TNF, MCP-1, IL-4, TGF-β1, and IL-10 using ELISA and for the identification of immunocontent markers of M1 and M2 macrophage phenotype using Western blotting.

A total of 88 animals were used in experimental protocols (Figures 1A,B). In each experiment, mice were randomly divided into 4 groups of 11 animals each. The following experimental groups were used in both protocols: (1) Saline/Sham, animals supplemented with saline (NaCl 0.9%) for 30 days and subsequently submitted to anesthesia (thiopental, 80 mg/kg) for 3 h without performing the CPIP model; (2) Saline/CPIP, animals supplemented with saline (NaCl 0.9%), subsequently anesthetized (thiopental, 80 mg/kg) and submitted to the CPIP model; (3) Corn Oil/CPIP, animals supplemented with corn oil (1,500 mg/kg), then anesthetized (thiopental, 80 mg/kg) and submitted to the CPIP model; (4) Omega-3/CPIP, animals supplemented with 1,500 mg/kg of omega-3, subsequently anesthetized (thiopental, 80 mg/kg) and submitted to the CPIP model.

The number of animals per group was determined using the sample size equation without replacement: *N* = {[(*z* alpha + z beta)*s]/sigma}^2^ (Daniel and Cross, 2018). The alpha and *z* alpha values were fixed at 0.05 and 1.96, respectively. The beta and *z* beta values were determined at 0.10 and 1.28, respectively. A minimum of 40% was established as the targeted difference value between the group means (sigma), based on previous experimental data (Belmonte et al., 2018) from our research group, and the standard deviation (*SD*) value was set at 35% related to the mean value. Thus, the obtained value is *N* = {[(1.96 + 1.28)*35]/40)}^2^ = 8 per group. Considering the exclusion criteria and possible loss of animals during a follow-up (death during the induction of the CPIP model or treatments, or if the animals presented signs of suffering during the experiment, such as self-mutilation, vocalization when stimulated, etc.), an increase in the group sample size of 30% was stipulated. Therefore, for each group of 8 animals required for adequate statistical power, 11 animals per group were submitted to the CPIP model.

### Measurement of Paw Edema

Paw edema was assessed by measuring the thickness of the middle portion of the right hind paw using a digital micrometer (Insize, Loganville, GA, United States). The measurement was carried out by positioning the device between the back and the plantar portion of the paw (Erthal et al., 2016). The results of paw thickness were expressed in micrometers (μm).

### Biochemical Assays

Following 30 days of omega-3, saline, or corn oil supplementation, and 48 h (protocol 2) or 15 days (protocol 1) after the induction of the CPIP model, the mice were anesthetized (1–2% isoflurane at 100% oxygen) and the euthanasia was performed by decapitation to dissect the skin and muscles of the right hind paw and perform the biochemical tests described below. Post dissection, the samples were immediately frozen in liquid nitrogen and stored in a freezer at -80°C until their analysis.

#### Enzyme-Linked Immunosorbent Assay

Enzyme-Linked Immunosorbent Assays were carried out on samples homogenized in phosphate-buffered saline (PBS), containing: Tween^®^ 20 (0.05%), PMSF (0.1 mM), EDTA (10 mM), aprotinin (2 ng/ml), and chloride benzethonium (0.1 mM) (Sigma-Aldrich, St. Louis, MO, United States). The samples were then centrifuged at 6,000 × g for 15 min (4°C), and the supernatant was collected and stored at -80°C for future analysis. The total protein content of the supernatant was measured by the Bradford method using a standard calibration curve with BSA (0.05–0.5 mg/ml) (Bradford, 1976). Aliquots with 100 μl were used to measure cytokine concentrations (IL-4, IL-10, TGF-β1, MCP-1, and TNF) using ELISA kits for mice (Invitrogen/Thermo Fisher Scientific, Waltham, MA, United States) according to the manufacturer’s instructions. Cytokine concentrations were measured by interpolating an 8-point standard curve with colorimetric assays at 450 nm (corrected by subtracting readings at 550 nm) on a plate spectrophotometer (Perlong DNM-9602, Nanjing Perlove Medical Equipment Co., Nanjing, China). The values were expressed as pg (cytokine) per mg (protein), as briefly described (Belmonte et al., 2018).

#### Western Blotting Assay

Western blotting assays were used to determine protein immunocontent: NOS-2 (or iNOS) and CD86 were the M1 macrophage markers and Arginase-1 was the M2 macrophage marker. Samples were homogenized and incubated in RIPA lysis buffer [1% Nonidet P-40, 0.5% sodium deoxycholate, 0.1% sodium dodecyl sulfate (SDS), and PBS] plus 100 mM sodium orthovanadate, 100 mM PMSF, and a cocktail of 1% protease inhibitors (Sigma-Aldrich, St. Louis, MO, United States). Then, the samples were incubated on ice for 30 min. After centrifugation at 6,000 × g for 20 min (at 4°C), the supernatant was collected, separated, and stored in a -80°C freezer. Protein content was measured using the Bradford method, using a standard calibration curve with BSA (0.05–0.5 mg/ml). Total protein aliquots (50 μg) were boiled at 95°C for 5 min in 25% volume in Laemmli buffer (1M sodium phosphate pH 7.0, 10% SDS, 10% β-mercaptoethanol, 50% glycerol, 0.1% bromophenol blue, Sigma-Aldrich, St. Louis, MO, United States).

The samples were submitted to electrophoresis on 8% polyacrylamide gel. Proteins were then transferred onto a nitrocellulose membrane for 2 h at 90 V constant voltage. The membrane was incubated for 1 h in blocking solution (5% milk powder, Molico^®^) and then washed and incubated with Ponceau S solution (Ponceau S solution, P7170, Sigma-Aldrich, MO, United States) for the determination of the transferred proteins. This mark was captured by colorimetric reading on a photo-documenter and used as a protein load control. The membranes were incubated overnight at 4°C with the primary antibodies after Ponceau S washing and stain removal: rabbit anti-iNOS (NOS-2, Cat. NBP1-33780, Novus Biologicals, Centennial, CO., United States); mouse anti-CD86 (Cat. Ab213044, Abcam, Cambridge, MA, United States); or rabbit anti-Arginase-1 (Cat. 93668S, Cell Signaling Technology, Danvers, MA, United States). After washing in Tris Buffer Saline with Tween^®^ 20 (TBS-T) (137 mM NaCl and 20 mM Tris HCl + 0.1% Tween^®^ 20, pH 7.6) the membranes were incubated with the specific secondary antibody was conjugated to peroxidase at room temperature for 1 h (Abcam, Cambridge, MA, United States). After this period, a new 30-min wash with TBS-T was performed, followed by exposure of the membranes for 1 min to the Chemiluminescence kit (ECL, Bio-Rad Laboratories, CA, United States) and detection using an imaging system (iBright Imaging Systems, Invitrogen/Thermo Fisher Scientific, Waltham, MA, United States). The quantification of protein bands was performed by densitometry using the iBright Analysis Software (Invitrogen/Thermo Fisher Scientific, Waltham, MA, United States). The values were graphically expressed as arbitrary units in relation to the control (Saline/Sham), as briefly described (Mazzardo-Martins et al., 2018).

### Statistical Analysis

Statistical analysis was performed by the Graph Pad Prism^®^ software, version 8.0 (La Jolla, CA, United States). Data distributions were assessed using the Shapiro–Wilk test. Data considered as parametric were presented as mean ± *SD*. Paw thickness measurement data were compared using a two-way analysis of variance (ANOVA) with repeated measurements, followed by the Tukey test. Independent variables were treatment (between-subject) and time (within-subject). The biochemical data were analyzed by one-way ANOVA, followed by Tukey’s multi-comparison test. The independent variable was treatment (between-subject). For all analyses, *p* < 0.05 was considered significant.

## Results

### Omega-3 Supplementation Prevents Paw Edema

**FIGURE 2:**
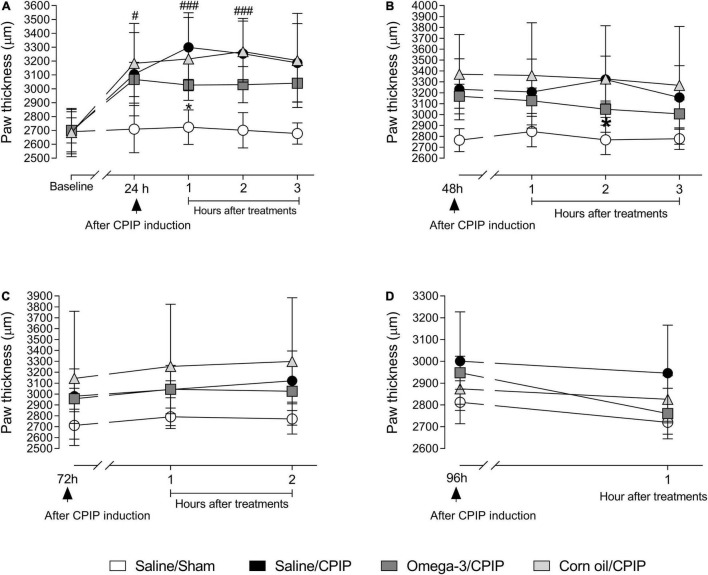
Effects of omega-3 supplementation on paw edema post-CPIP induction. Paw thickness evaluations of animals in Saline/Sham (*n* = 10), Saline/CPIP (*n* = 9), Omega-3/CPIP, and Corn oil/CPIP (*n* = 10) groups 1 day before the induction of the CPIP model (baseline, **A**), and time course of the saline, omega-3, or corn oil treatments on days 1, 2, 3, and 4 after model induction **(A–D)**. Assessments at 24 **(A)** and 48 h **(B)** post-injury, and 1, 2, and 3 h post-treatment. Time course up to 2 h post-treatment, 72 h after the induction of CPIP **(C)**. Paw thickness at 96 h post-CPIP and 1 h post-treatment **(D)**. Data are expressed as mean ± *SD* compared using the two-way ANOVA with repeated measurements followed by Tukey’s test. ^#^*p* < 0.05 and ^###^*p* < 0.0001 vs. Saline/Sham group; *p < 0.05 vs. Saline/CPIP group.

The CPIP model induced edema, as observed by the increased paw thickness of animals in the Saline/CPIP group at 24, 48, and 72 h post-injury, compared to the Saline/Sham group (Figures 2A–D). Paw thickness returned to baseline values on the 4th-day post-injury.

Paw thickness was significantly altered by treatment at 24 h post-injury [*F*(4, 140) = 7.11, *p* = 0.01] (Figure 2A). Planned group comparisons indicated that supplementation with omega-3 reduced the paw thickness (i.e., edema) of animals submitted to CPIP (the Omega-3/CPIP group) in the 1st h after acute post-CPIP omega-3 treatment, compared to the control group (the Saline/CPIP group) (*p* < 0.05). Although the Omega-3/CPIP group was not statistically different from the Saline/CPIP group at the two other assessment points on day 1 post-CPIP, the mean paw thickness remained directionally lower in this group up to the 3rd h after treatment (Figure 2A). Paw thickness was also altered significantly by treatment on the 2nd day post-CPIP [*F*(3, 105) = 5.37, *p* = 0.02] (Figure 2B). Omega-3 supplementation significantly decreased the paw thickness of animals in the 2nd h after acute omega-3 treatment when compared to the Saline/CPIP group (*p* < 0.05). Also, as on the 1st-day post-CPIP, mean paw thickness on the 2nd-day post-CPIP remained directionally lower in the Omega-3/CPIP group up to the 3rd h after acute omega-3 administration but with no statistically significant difference compared to the Saline/CPIP group (Figure 2B).

Figures 2C,D show paw thickness assessment at 72 and 96 h post-CPIP induction, respectively. The statistical differences between Saline/CPIP and Omega-3/CPIP groups noted at 1-day and 2-day post-CPIP were no longer observed (i.e., no acute effects of omega-3 supplementation). The within-subject time factor showed a significant effect at 72 h post-CPIP induction [*F*(2, 70) = 11.51, *p* < 0.0001], reflecting increased edema over time. Also, the treatment factor showed a significant effect 72 h post-CPIP [*F*(2, 70) = 4.49, *p* = 0.04]. Finally, at 96 h post-induction, a significant interaction between time and treatment was noted [*F*(1, 34) = 6.53, *p* = 0.01, Figure 2D). Visual inspection of Figure 2D suggested that this interaction was due in part to relatively stable levels of edema over time in the Saline/CPIP group, with the Omega-3/CPIP group showing decreased edema over time. However, *post hoc* between-group comparisons showed no statistical differences between these two groups. In the 1st h after the treatment with omega-3 at 96 h post-CPIP, the Omega-3/CPIP group presented a mean thickness of 2,760 ± 132.6 μm, returning to baseline values, and similar to the paw thickness observed in the Saline/Sham group (2,812.3 ± 49.31 μm, Figure 2D).

The Corn Oil/CPIP group was used as an additional control in our study, reflecting high omega-6 but low omega-3 content. The analysis showed that there was no difference between the Saline/CPIP and Corn Oil/CPIP groups (Figures 2A–D). Both the groups increased paw thickness relative to baseline at all times assessed and returned to their baseline values at 96 h post-injury (Figure 2D). Nonetheless, there were no significant differences between Corn Oil/CPIP and Omega-3/CPIP groups despite the latter group showing directionally lower edema throughout the protocol.

### Omega-3 Supplementation Does Not Alter Pro-inflammatory Cytokine Concentrations in the Paw Skin and Muscle at 48 h Post-chronic Post-ischemia Pain

**FIGURE 3:**
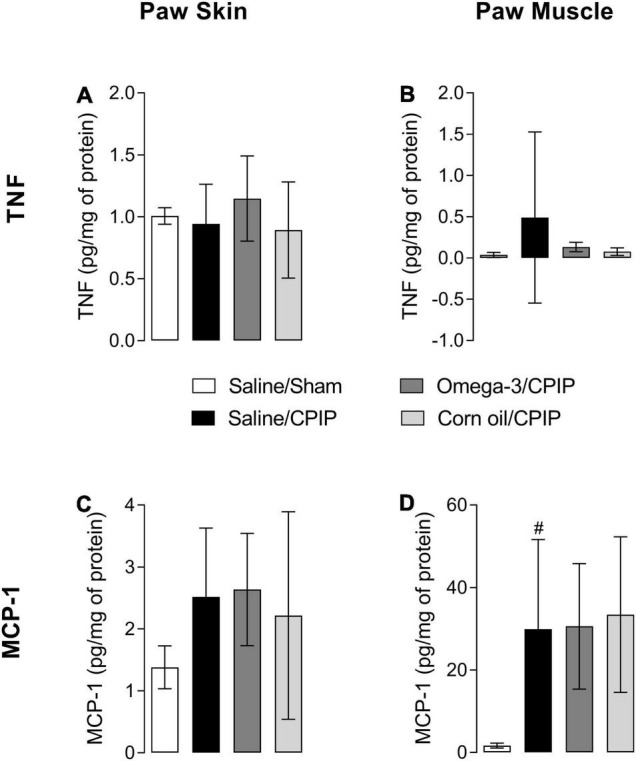
Effects of omega-3 supplementation on tumor necrosis factor (TNF) and monocyte chemotactic protein-1 (MCP-1) concentrations in the paw skin and muscle of mice 48 h after the induction of the CPIP model. TNF concentrations in the skin **(A)** and muscle **(B)** of Saline/Sham, Saline/CPIP, Omega-3/CPIP, and Corn oil/CPIP groups. MCP-1 concentrations in the skin **(C)** and muscle **(D)** of Saline/Sham, Saline/CPIP, Omega-3/CPIP, and Corn oil/CPIP groups. Data are expressed as mean ± *SD* of 6–7 animals per group, statistically assessed by the one-way ANOVA followed by Tukey’s test. ^#^*p* < 0.05 vs. the Saline/Sham group.

Tumor necrosis factor levels were not changed significantly in the paw skin and muscle of animals submitted to CPIP (Saline/CPIP group) when compared to the Saline/Sham group (Figures 3A,B). Omega-3 supplementation also did not alter skin TNF levels of injured animals (Omega-3/CPIP group) when compared to injured animals that received saline or corn oil (Figure 3A). Directional reductions in TNF muscle concentration in the Omega-3/CPIP group and the Corn oil/CPIP group relative to the Saline/CPIP group did not reach statistical significance, in part due to high TNF variability in the Saline/CPIP group (Figure 3B).

The concentration of MCP-1 in the paw skin did not change significantly with CPIP injury across groups (Figure 3C). However, a significant increase of MCP-1 in the paw muscle of animals in the Saline/CPIP group was observed when compared to the Saline/Sham group (*p* < 0.05) (Figure 3D). Nonetheless, omega-3 supplementation did not change the concentration of MCP-1 in the paw muscle or skin when Saline/CPIP and Omega-3/CPIP groups were compared (Figures 3C,D). Similarly, the group supplemented with corn oil (Corn oil/CPIP group) did not show significant changes in the levels of the pro-inflammatory cytokines TNF and MCP-1 in the paw skin or muscle when compared to Saline/CPIP or Omega-3/CPIP groups (Figures 3C,D).

### Omega-3 Supplementation Alters Anti-inflammatory Cytokine Concentrations in the Paw Muscle at 48 h Post-chronic Post-ischemia Pain

**FIGURE 4:**
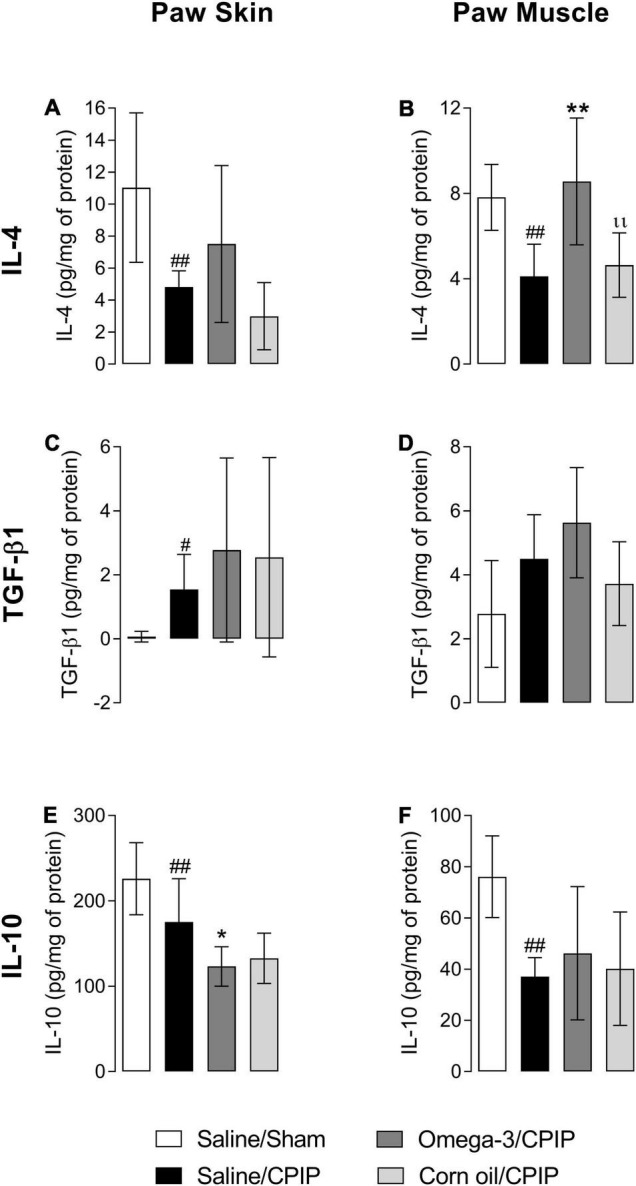
Effects of omega-3 supplementation on the concentrations of interleukin- (IL-) 4, transforming growth factor-β1 (TGF-β1), and IL-10 in the skin and muscle of mice 48 h after the induction of the CPIP model. IL-4 concentrations in the skin **(A)** and muscle **(B)** of Saline/Sham, Saline/CPIP, Omega-3/CPIP, and Corn oil/CPIP groups. TGF-β1 concentrations in the skin **(C)** and muscle **(D)** of Saline/Sham, Saline/CPIP, Omega-3/CPIP, and Corn oil/CPIP groups. IL-10 concentrations in the skin **(E)** and muscle **(F)** of Saline/Sham, Saline/CPIP, Omega-3/CPIP, and Corn oil/CPIP groups. Data are expressed as mean ± *SD* of 6–7 animals per group, statistically assessed by the one-way ANOVA followed by Tukey’s test. ^#^*p* < 0.05 and ^##^*p* < 0.01 vs. the Saline/Sham group; *p < 0.05 and **p < 0.01 vs. the Saline/CPIP group. ıı *p* < 0.01 vs. the Omega-3/CPIP group.

Figures 4A, B shows that CPIP injury reduced the concentration of IL-4 in both the skin (*p* < 0.01) and muscle (*p* < 0.01) of animals in the Saline/CPIP group when compared to the Saline/Sham group. Although omega-3 supplementation did not change the concentration of IL-4 in the paw skin when compared to the Saline/CPIP group (Figure 4A), omega-3 supplementation did prevent the reduction of IL-4 in the paw muscle, with the observation of a significant difference when compared to Saline/CPIP (*p* < 0.01) or Corn oil/CPIP (*p* < 0.01) groups (Figure 4B).

Transforming growth factor-β1 concentrations increased in the paw skin of the Saline/CPIP group when compared to the Saline/Sham group (*p* < 0.01, Figure 4C). However, CPIP did not significantly alter the concentrations of this cytokine in the paw muscle (Figure 4D). In the Omega-3/CPIP group, there was a small increase in TGF-β1 levels in the skin and muscle, but this change was not significant when compared to the Saline/CPIP group (Figures 4C,D). Figure 4E shows a significant decrease in IL-10 concentrations in the paw skin of the Saline/CPIP group when compared to the Saline/Sham group (*p* < 0.01), with similar changes noted in the muscle (*p* < 0.01, Figure 4F). Omega-3 supplementation unexpectedly decreased IL-10 concentrations in the paw skin compared to the Saline/CPIP group (*p* < 0.05, Figure 4E), whereas IL-10 levels in the Omega-3/CPIP group remained statistically similar to the Saline/CPIP group in the paw muscle (Figure 4F).

Treatment with corn oil (Corn oil/CPIP group) did not alter the concentrations of IL-4, TGF-β1, or IL-10 in the paw skin (Figures 4A,C,E) or muscle (Figures 4B,D,F) when compared to Saline/CPIP or Omega-3/CPIP groups.

### Omega-3 Supplementation Does Not Alter the Macrophage Phenotype in the Paw Muscle at 48 h Post-chronic Post-ischemia Pain

**FIGURE 5:**
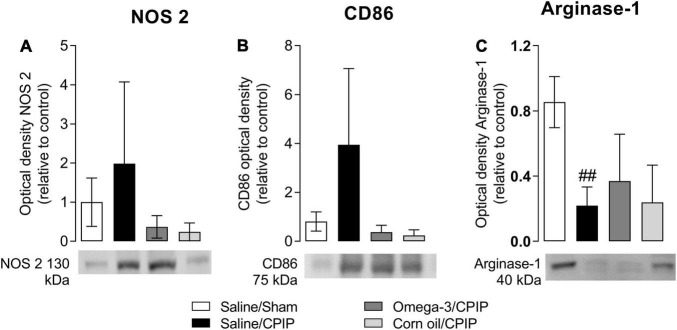
Effects of omega-3 supplementation on the immunocontent of macrophages M1 markers: nitric oxide synthase 2 (NOS-2) **(A)** and CD86 **(B)**, and M2 marker: Arginase-1 **(C)** on the mouse paw muscle 48 h after the model induction in Saline/Sham, Saline/CPIP, Omega-3/CPIP, and Corn oil/CPIP groups. Data are expressed as mean ± *SD* of 6 animals per group, statistically assessed by the one-way ANOVA followed by Tukey’s test. ^##^*p* < 0.01 vs. the Saline/Sham group.

The immunocontent of NOS-2 and CD86 (phenotype M1 marker) and Arginase-1 (phenotype M2 marker) on the 2nd day after the induction of the CPIP model is shown in Figure 5. Ischemia/reperfusion (I/R) injury (Saline/CPIP) did not significantly alter the immunocontent of NOS-2 (*p* = 0.7409, Figure 5A) or CD86 (*p* = 0.1390, Figure 5B) markers compared to the Saline/Sham group. However, I/R injury reduced the immunocontent of Arginase-1 (*p* = 0.0011, Figure 5C) in the paw muscle of animals in the Saline/CPIP group at 48 h post-CPIP when compared to the Saline/Sham group. Preventive supplementation with omega-3 (Omega-3/CPIP) did not significantly alter the immunocontent of NOS-2 (*p* = 0.4014, Figure 5A), CD86 (*p* > 0.999, Figure 5B), or Arginase-1 (*p* = 0.6365, Figure 5C) compared to the Saline/CPIP group. The control treatment with corn oil also did not alter the immunocontent of NOS-2 (Figure 5A), CD86 (Figure 5B), or Arginase-1 (Figure 5C) compared to Saline/CPIP or Omega-3/CPIP groups.

### Omega-3 Supplementation Increases IL-10 Concentration in the Paw Muscle at 15 Days Post-chronic Post-ischemia Pain

**FIGURE 6:**
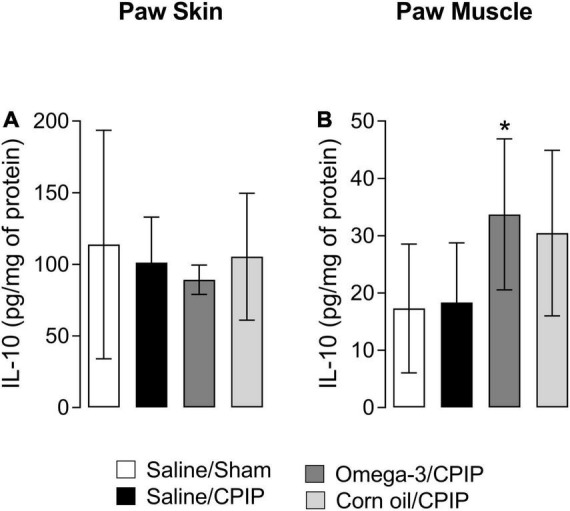
Interleukin-10 concentrations in the paw skin and muscle 15 days after the induction of the CPIP model. IL-10 concentrations in the skin **(A)** and muscle **(B)** of Saline/Sham, Saline/CPIP, Omega-3/CPIP, and Corn oil/CPIP groups. Data are expressed as mean ± *SD* of 6–7 animals per group, assessed by the one-way ANOVA followed by Tukey’s test. *p < 0.05 vs. the Saline/CPIP group.

The IL-10 concentration in the paw skin and muscle of animals from the Saline/CPIP group was not significantly altered in relation to the Saline/Sham group 15 days after the induction of the CPIP model (Figures 6A,B). Omega-3 supplementation also did not change the IL-10 concentration in the paw skin at 15 days post-CPIP when compared to the Saline/CPIP group (Figure 6A). However, omega-3 supplementation significantly increased anti-inflammatory IL-10 concentrations in the paw muscle when compared to the Saline/CPIP group (*p* = 0.0378, Figure 6B). The control treatment with corn oil (Corn oil/CPIP group) did not alter IL-10 concentrations in the paw skin or muscle when compared to either Saline/CPIP (*p* = 0.1072) or Omega-3/CPIP (0.6429) groups (Figures 6A,B).

## Discussion

Several clinical (Lee et al., 2012; Abdulrazaq et al., 2017; Gioxari et al., 2018) and preclinical (Yoshino and Ellis, 1987; Reddy and Lokesh, 1994; Wohlers et al., 2005; Nobre et al., 2013; Torres-Guzman et al., 2014; Lobo et al., 2016) studies have defined the benefits of omega-3 supplementation in the prevention and treatment of inflammatory diseases. However, there is a lack of evidence regarding the effects of omega-3 supplementation on CRPS-I. Omega-3 effects on immunoregulatory mechanisms in CPIP animal models of CRPS-I have not been studied although such studies appear warranted given the involvement of inflammatory mechanisms in early CRPS (Bruehl, 2015; Bruehl et al., 2016). This is the first study to evaluate the effects of preventive omega-3 supplementation (for 30 days) on edema-related inflammatory process, pro- and anti-inflammatory cytokine regulation, and the macrophages’ phenotypic characteristics present at the injury site in CPIP mice. The dose of omega-3 used was defined based on previous studies that used omega-3 in animal models of inflammatory and neuropathic pain (Nobre et al., 2013; Redivo et al., 2016, 2019; Silva et al., 2017). Also, we used corn oil as a control supplement due to its inactive state in terms of anti-inflammatory effects and its high omega-6 rather than omega-3 content (Pérez et al., 2005; Freitas et al., 2016).

Complex regional pain syndrome resulting from ischemia and reperfusion causes an intense inflammatory process and edema, with the latter being one of the most common clinical signs of CRPS besides pain (Bruehl et al., 2002; Coderre et al., 2004; Bruehl, 2015; Birklein and Dimova, 2017). Here, we observed that the animal model of CPIP promoted paw edema consistent with that often occurring in CRPS that remained from the 1st to the 3rd-day post-injury. Coderre et al. (2004) demonstrated that injury-related edema occurred 3–4 h after the paw injury although it was noted to remit after 24 h. On the other hand, Klafke et al. (2016) did not observe edema in rats after the induction of CPIP as long as 14 days post-CPIP. This variability in edema in the CPIP model seems to be in parallel that edema is not always present in humans suffering from CRPS. Rather, it can be short-lived, variable, or prolonged for months or years (Bruehl et al., 2002). When present in preclinical studies, edema may last for different durations depending on the species of the animal, lineage, sex, weight, age, and even procedure standardization, and it may occur from the 1st to the 3rd-day post-CPIP (Tang et al., 2017; Mazzardo-Martins et al., 2018; De Prá et al., 2019).

In this study, omega-3 preventive supplementation reduced the paw edema of animals with CPIP. Few studies in the literature have demonstrated the anti-edematogenic effects of fish-derived omega-3 fatty acids, including EPA and/or DHA (Yoshino and Ellis, 1987; Reddy and Lokesh, 1994; Wohlers et al., 2005; Nobre et al., 2013; Torres-Guzman et al., 2014; Lobo et al., 2016), with none using a CRPS-relevant model. In the carrageenan-induced paw edema model of inflammatory pain, acute treatment with omega-3 (at doses of 1, 2.5, and 5 mg/kg, i.g., much lower doses than in the current study) reduced the paw edema of rats (Nobre et al., 2013), with similar findings in chronic treatment with different fish oils added to the standard diet (Yoshino and Ellis, 1987; Reddy and Lokesh, 1994; Wohlers et al., 2005). A study using the complete Freund’s adjuvant- (CFA-) induced arthritic rat model similarly demonstrated that i.g., chronic treatment with DHA (at doses of 10, 30, and 100 mg/kg) reduced the edema of the knee joint (Torres-Guzman et al., 2014). Finally, it was demonstrated that pure and concentrated fish oil reduced paw edema by approximately 14%, 48 h after the induction of the intra-plantar CFA model (Lobo et al., 2016). These studies suggest that omega-3 PUFA decreases the levels of arachidonic acid in immune cells. As a consequence, the enzymes cyclooxygenases and lipoxygenases have EPA and DHA as a substrate, inhibiting the production of inflammatory eicosanoids such as PG (PGE2) and leukotrienes (LTB4), which are responsible for edema formation during the inflammatory process (Reddy and Lokesh, 1994; Nobre et al., 2013; Torres-Guzman et al., 2014; Lobo et al., 2016). Also, NO—which is mainly produced by M1 macrophages—causes vasodilation and vascular permeability in some models (e.g., carrageenan-induced inflammation), and omega-3 PUFA is able to decrease the NO levels, evaluated by nitrite production (Wohlers et al., 2005).

Inflammatory mechanisms leading to edema may differ somewhat in the CPIP model. Following I/R injury, there is a significant increase in the formation of reactive oxygen species, oxidative stress, and activation of resident immune cells (mainly macrophages). More specifically, there is increased activity in pro-inflammatory cytokine signaling pathways involving TNF, IL-1β, and IL-6 in the paw skin and muscle (Laferrière et al., 2008; Hsiao et al., 2016; Klafke et al., 2016; Hewedy, 2018; Santos et al., 2021). These changes are typical of early CRPS-I in humans, causing induction and regulation of the inflammatory response and contributing to neurogenic inflammation (Laferrière et al., 2008; Marinus et al., 2011; Klafke et al., 2016; Hewedy, 2018). The present study examined the concentrations of pro-inflammatory cytokines TNF and MCP-1 and anti-inflammatory IL-4, IL-10, and TGF-β1 in the paw skin and muscle 48 h after the induction of CPIP in mice previously supplemented with omega-3, corn oil, or saline. The concentrations of TNF in the paw skin and muscle in mice from the Saline/CPIP group at 2 days post-CPIP were not significantly changed compared to the Saline/Sham group. In a prior study using the CPIP model, TNF concentrations were found to be increased 1, 2, and 7 days post-I/R in the skin and muscle of the paw (Hsiao et al., 2016; Santos et al., 2021) although this study was conducted in male rather than female Swiss mice. On the other hand, Laferrière et al. (2008) observed in male rats that TNF levels were increased at 2 h post-injury and returned to baseline levels on the 2nd-day post-I/R, consistent with our findings. There are no prior studies of CPIP effects on TNF in female Swiss mice available to provide context for our findings. Our results also showed that although the concentration of MCP-1 did not change in the paw skin, it was increased in the muscle of animals undergoing CPIP (with saline). The concentration of this cytokine in the skin and muscle of the paw post-I/R injury has not been reported in prior research. However, an animal model of CRPS that used the transfer of purified serum immunoglobulin G (IgG) from human patients with longstanding CRPS into mice (in addition to plantar skin and muscle incision) showed that MCP-1 concentrations in the paw skin were increased from 1 to 3 days following injury, as in the current study, but were normalized at 6 and 13 days after model induction (Helyes et al., 2019).

Our study demonstrated that omega-3 directionally reduced TNF levels in the muscle post-CPIP although this effect was not significant due in part to the high variability of TNF levels in the saline/CPIP group. Although omega-3 effects on TNF were not statistically significant, it is nonetheless possible that even the modest reductions observed could have clinical significance as TNF is one of the main cytokines involved in initiating the immune and inflammatory cascade (Galley and Webster, 1996). Even when released at low concentrations, TNF activates endothelial cells promoting vasodilation, contributing to the formation of edema, and stimulating the secretion of chemokines such as MCP-1 observed to be increased in CPIP animals. MCP-1 recruits monocytes to the injury site, in addition to activating them for a pro-inflammatory macrophage phenotype (Mantovani et al., 2004). Lobo et al. (2016) demonstrated that the treatment with pure fish oil (EPA at 460 mg and DHA at 200 mg) or concentrate (EPA at 460 mg and DHA at 360 mg or EPA at 690 mg and DHA at 540 mg) 7 days before and 5 days after intra-plantar CFA-induced inflammation decreased TNF levels in the paw skin of male and female rats. Another study using orally administered omega-3 at single doses of 2.5 or 5 mg/kg showed a decrease in TNF levels in the paw of mice submitted to the carrageenan-induced edema model (Nobre et al., 2013). A study by our group (Belmonte et al., 2018) evaluated the concentration of pro- and anti-inflammatory cytokines in the spinal cord of male Swiss mice 11 days after the induction of the CPIP model and found no increase in spinal cord TNF, IL-6, or IL-10 levels although IL-1β concentrations were elevated. Importantly, that study did not assess concentrations at the site of I/R injury as assessed in this study. Human studies evaluating the serum protein or blood mRNA levels of patients with CRPS-I have shown that soluble TNF receptors 1 and 2 (sTNFR1 and sTNFR2) and inflammatory cytokines such as TNF, IL-2, and IL-8 are increased relative to non-CRPS controls (Schinkel et al., 2006; Uçeyler et al., 2007). In a prior study addressing the changes in cytokine levels specifically in the affected limb as in the current study, blister fluid obtained from the affected extremities of patients with CRPS-Type I showed increased IL-6, IL-8, TNF, IL-12p40/p70, MCP-1, and macrophage inflammatory protein- (MIP-) 1β levels relative to the contralateral limb (Heijmans-Antonissen et al., 2006).

To date, little is known regarding the anti-inflammatory cytokines in the CPIP model of CRPS although some human work suggests that differences might be expected. For example, a clinical study evaluated 42 patients with CRPS-Type I and 34 healthy controls and found that systemic anti-inflammatory IL-4 and IL-10 mRNA and protein levels were reduced in patients with CRPS. In contrast, TGF-β1 mRNA levels did not differ between groups although TGF-β1 protein levels were reduced in patients with CRPS relative to controls (Uçeyler et al., 2007). Other study indicates that IL-10 levels in blister fluid of patients with CRPS-Type I were similar in involved and non-involved extremities (Heijmans-Antonissen et al., 2006). Consistent with the abovementioned human clinical findings regarding systemic anti-inflammatory cytokine mRNA and protein levels, our results in the CPIP model demonstrated that I/R-induced injury decreased IL-4 and IL-10 in the paw skin and muscle. However, in contrast to human clinical findings, we found that TGF-β1 was increased in the skin of CPIP animals. Omega-3 considerably increased anti-inflammatory IL-4 concentrations in the muscle but did not increase IL-10 or TGF-β1 concentrations at 2 days post-injury. Surprisingly, omega-3 supplementation further reduced IL-10 levels in the paw skin compared to the saline group when assessed at 2-day post-CPIP. However, this pattern changed over time. Omega-3 supplementation significantly increased IL-10 levels in the paw muscle at 15 days post-CPIP.

Prior study has not examined whether CPIP alters anti-inflammatory cytokines or examined what effect omega-3 supplementation might have. It may, therefore, be useful to consider related literature as a context for our findings regarding omega-3 effects on IL-10. The most relevant finding is that C57BL/6N mice receiving a diet rich in DHA (Linoleic 2.9 g/kg, Linolenic 0.43 g/kg, and DHA 0.26 g/kg) for 7 days before myocardial I/R displayed decreased IL-10 mRNA expression 72 h after reperfusion, similar to our findings at 2 days post-CPIP (Habicht et al., 2020). Furthermore, DHA supplementation also reduced TNF and IL-1β expression at 72 h post-I/R injury and was associated with a strong increase in the phenotype of alternatively activated Ly6C + macrophages at 14 days post-injury (Habicht et al., 2020). In contrast, mice with hemorrhagic cystitis acutely treated with DHA at 1 μmol/kg (i.p.) did not show altered levels of IL-10 (Freitas et al., 2016). The results of a prior study focused on specialized pro-resolving mediators derived from omega-3 fatty acids, such as resolvin D1 and Maresin-1 (MaR1), following I/R injury appear discrepant. Resolvin D1 (100 μg/kg, intravenous) did not inhibit an increase in IL-10 post lung I/R injury (Zhao et al., 2016), and MaR1 (4 ng/g body weight) administered prior to ischemia in a rat model of liver I/R injury increased serum IL-10 levels that were otherwise reduced post-I/R injury (Soto et al., 2020). Although existing data are sparse and somewhat mixed, our findings suggest that the beneficial effects of omega-3 may depend on the modulation of the long-term inflammatory response initiated by the I/R injury and omega-3 supplementation may help balance the pro- and anti-inflammatory states, resulting in reduced inflammation.

The current study also considered macrophage phenotypes relevant to inflammation. On the 2nd day post-I/R injury, significant reductions were observed in Arginase-1 immunocontent, which is an M2 macrophage phenotype immunomarker. In contrast, NOS-2 and CD86 (M1 macrophage phenotype immunomarkers) content showed a tendency to increase in the Saline/CPIP group, suggesting a predominance of M1 macrophages in the muscle post-injury. We found that omega-3 supplementation did not alter the macrophage phenotype at 48 h post-injury. This finding is confirmed by the observed pattern of evaluated cytokines and reinforces the prior literature demonstrating that M2 macrophages produce high levels of IL-10 in comparison with M1 macrophages (Verreck et al., 2004).

Interleukin-4, which was significantly elevated at 2 days post-CPIP by omega-3 supplementation in the current study, is a crucial cytokine for inducing polarization of M1 to M2 phenotype macrophages. In addition, IL-4 is involved in tissue repair and healing (Mokarram et al., 2012) and can also act on peripheral neuronal terminals, contributing to the balance between facilitating and inhibiting inflammatory pain (Mousa et al., 2004). M2 macrophages are induced in response to Th2 cytokines. Based on different contexts, we observe the different subtypes M2a, M2b, M2c, and M2d. These subtypes have immunoregulatory activities capable of secreting high levels of anti-inflammatory mediators such as IL-10 and low levels of pro-inflammatory cytokines, such as IL-12 (Mantovani et al., 2004; Vogel et al., 2014; Iqbal and Kumar, 2015). They can also produce high levels of IL-8, MCP-1, MIP-1β, etc., to recruit neutrophils, monocytes, and T-lymphocytes in an anti-inflammatory or a regulatory response (Verreck et al., 2004, 2006).

We hypothesize that the observed omega-3 induced elevations in IL-4 levels in the muscle on the 2nd-day post-CPIP-induced late macrophage polarization for the M2 phenotype. These changes, in turn, resulted in subsequent increases in IL-10 production long in post-CPIP injuries, like those observed in the current work at 15-day post-CPIP. Although we were unable to assess macrophage phenotypes at 15-day post-CPIP due to financial constraints, our findings are in agreement with Burger et al. (2019), who observed an acceleration in the healing process following EPA supplementation for 4 weeks, with these changes in parallel with an increase in M2 macrophages and IL-10 on the 7th day after the induction of skin wounds in mice. Based on these findings, we hypothesize that omega-3 supplementation might help shift the body toward a more anti-inflammatory phenotype following the injuries that are known to trigger CRPS-I.

This study has some limitations that should be addressed in future research. Unfortunately, we did not measure the levels of other pro- or anti-inflammatory cytokines, and we did not assess the macrophage phenotype at a 15-day post-I/R injury. Further studies are necessary to confirm the consistent omega-3 anti-inflammatory and pro-resolving effects at a later stage of the CPIP model. The current study is also limited by the absence of pain-specific outcomes, which are also a cardinal feature of CRPS. These results will be separately reported. In conclusion, our results demonstrate an apparent immunoregulatory effect of preventive omega-3 supplementation in an animal model of CRPS-I. The findings presented here expand upon and corroborate limited data in the existing literature, indicating that omega-3 supplementation has anti-edematogenic actions and beneficially regulates important anti-inflammatory cytokines, such as IL-4 (early) and IL-10 (late), in a CPIP model of CRPS-I.

## Data Availability Statement

The raw data supporting the conclusions of this article will be made available by the authors, without undue reservation.

## Ethics Statement

The animal study was reviewed and approved by the Ethics Committee on the Use of Animals (CEUA) from the University of Southern Santa Catarina (UNISUL), with protocol no. 18.050.4.01.IV.

## Author Contributions

FB and DFM: study conception and design. PFF, TOG, HSB, and VVH: acquisition of data. FB, JMM, APF, and DFM: analysis and interpretation of data. FB, JMM, SB, and DFM: drafting of manuscript. FB, DFM, SB, EBB, LAS, and ASIS: critical revision. All authors read and approved the final manuscript.

## Conflict of Interest

The authors declare that the research was conducted in the absence of any commercial or financial relationships that could be construed as a potential conflict of interest.

## Publisher’s Note

All claims expressed in this article are solely those of the authors and do not necessarily represent those of their affiliated organizations, or those of the publisher, the editors and the reviewers. Any product that may be evaluated in this article, or claim that may be made by its manufacturer, is not guaranteed or endorsed by the publisher.
